# Decoding maize drought tolerance: The role of the ZmSK1-ZmCPP2-ZmSOD4 module

**DOI:** 10.1093/plcell/koaf030

**Published:** 2025-02-10

**Authors:** Jiajun Wang

**Affiliations:** Assistant Features Editor, The Plant Cell, American Society of Plant Biologists, Rockville, MD 20850, USA; School of Life Sciences, Xiamen Key Laboratory of Plant Genetics, Xiamen University, Xiamen 361102, China

In recent years, global drought challenges have intensified significantly. Drought is one of the most significant and frequently encountered abiotic stresses impacting plant growth and development, causing substantial harm to physiological processes and substantially reducing crop yields (Kapoor et al. 2020). According to a United Nations report (Vicente-Serrano et al. 2024), more than three-quarters of the Earth's land has become drier over the past 30 years, with arid regions expanding by approximately 4.3 million square kilometers. Projections indicate that by 2040, climate change–induced drought expansion, amounting to a 3.9% increase in arid areas, could lead to the loss of approximately 20 million tons of maize, 19 million tons of rice, 8 million tons of soybeans, and 21 million tons of wheat.

Under drought conditions, metabolic imbalances lead to increased production of reactive oxygen species, disrupting cellular redox homeostasis. Thus, redox regulation and antioxidant systems are crucial for enhancing drought tolerance. The excessive accumulation of superoxide radicals (**·**O_2_⁻) during drought marks the initial stage of oxidative damage in plants. Superoxide dismutase (SOD), a group of metalloenzymes, catalyzes the dismutation of 2 **·**O_2_⁻ molecules and 2 protons (H^+^) into oxygen (O_2_) and hydrogen peroxide (H_2_O_2_) (Laxa et al. 2019). This enzymatic activity mitigates acute cellular damage and preserves membrane integrity, underscoring SOD's pivotal role in plant defense against drought-induced oxidative stress. Glycogen synthase kinase 3-like (GSK3-like) kinases also play a key role in enabling plants to adapt to various abiotic stresses, including drought (Song et al. 2023). However, the mechanisms by which GSK3-like kinases regulate drought resistance through antioxidant defense pathways remain unclear.

In new work, Yang Xiang and collaborators (Xiang et al. 2025) identified a pathway involving the GSK3-like kinase ZmSK1 along with the transcription factor cysteine-rich polycomb-like protein 2 (ZmCPP2) and superoxide dismutase 4 (ZmSOD4) as a key mechanism contributing to drought resistance in maize. The authors demonstrated that ZmSK1 acts as a negative regulator of antioxidant defense. Overexpression of *ZmSK1* decreased drought tolerance in maize, while the *zmsk1* loss-of-function mutant exhibited enhanced drought tolerance. In *ZmSK1*-overexpressing plants, oxidative damage indicators such as malondialdehyde content and electrolyte leakage were elevated, and SOD activity was reduced; conversely, these parameters were reversed in the *zmsk1* mutant. *ZmSOD4-*overexpressing plants exhibited reduced oxidative damage, increased SOD activity, and enhanced drought tolerance, opposite to the phenotypes of *ZmSOD4* RNAi plants.

To investigate the mechanism by which ZmSK1 suppresses antioxidant defense and negatively impacts drought tolerance, a yeast 2-hybrid screen was conducted to identify ZmSK1-interacting proteins. This led to the identification of ZmCPP2, a member of the CPP family, also known as the tesmin/TSO1-like CXC (TCX) protein family. In *Arabidopsis thaliana*, the homologous genes AtCPP4/SOL1/TCX3 and AtCPP6/SOL2/TCX2 redundantly regulate stomatal cell division, impacting stomatal development (Simmons et al. 2019) and suggesting a potential role in controlling water loss. The authors used a range of in vitro and in vivo assays, including pull-down, co-immunoprecipitation, luciferase complementation imaging, and bimolecular fluorescence complementation, to demonstrate that ZmSK1 and ZmCPP2 interact within the nucleus. Unlike *ZmSK1*-overexpressing plants, *ZmCPP2*-overexpressing plants showed reduced oxidative damage, increased SOD activity, and improved drought tolerance. Analysis of the drought tolerance phenotype of the *zmsk1 zmcpp2* double mutant revealed a significant reduction in drought tolerance compared with the wild type, closely resembling the phenotype of the *zmcpp2* mutant. These findings provide genetic evidence that ZmCPP2 functions epistatically to ZmSK1 in regulating drought tolerance in maize.

In vitro and in vivo phosphorylation assays demonstrated that ZmSK1 phosphorylates serine 250 of ZmCPP2, and the phosphorylation level of this site is reduced under drought conditions, likely due to the suppression of ZmSK1 kinase activity. Overexpression of *ZmCPP2^S250A^* enhanced drought tolerance in maize compared with *ZmCPP2^WT^*, whereas overexpression of *ZmCPP2^S250D^* reduced drought tolerance relative to *ZmCPP2^WT^*, although it still exhibited slightly greater tolerance than the wild type. Yeast 1-hybrid, electrophoretic mobility shift assay, and chromatin immunoprecipitation followed by quantitative PCR assays showed that ZmCPP2 directly binds to the ATTAAAATTTTGAAG element in the *ZmSOD4* promoter. Dual luciferase assay further confirmed that ZmCPP2 activates *ZmSOD4* transcription in tobacco leaves. Additionally, the transcription level of *ZmSOD4* was elevated in *ZmCPP2* overexpression lines and decreased in the *zmcpp2* mutant.

Further investigation revealed that serine 250 of ZmCPP2 is located on a loop following the CXC domain, where it interacts with the second T of the ATTAAAATTTTGAAG element in the *ZmSOD4* promoter. Phosphorylation at this site inhibits ZmCPP2's ability to bind the *ZmSOD4* promoter, though it does not affect its transcriptional activation activity at the C terminus. ZmSK1 significantly inhibits ZmCPP2's binding to the *ZmSOD4* promoter and reduces its transcription.

In conclusion, the authors present strong experimental evidence indicating that drought stress suppresses ZmSK1 kinase activity, thereby preventing the phosphorylation of serine 250 in ZmCPP2, which enhances its binding to the *ZmSOD4* promoter to promote *ZmSOD4* expression, elevates SOD activity, and ultimately improves the plant's drought resistance (Fig.).

**Figure.**
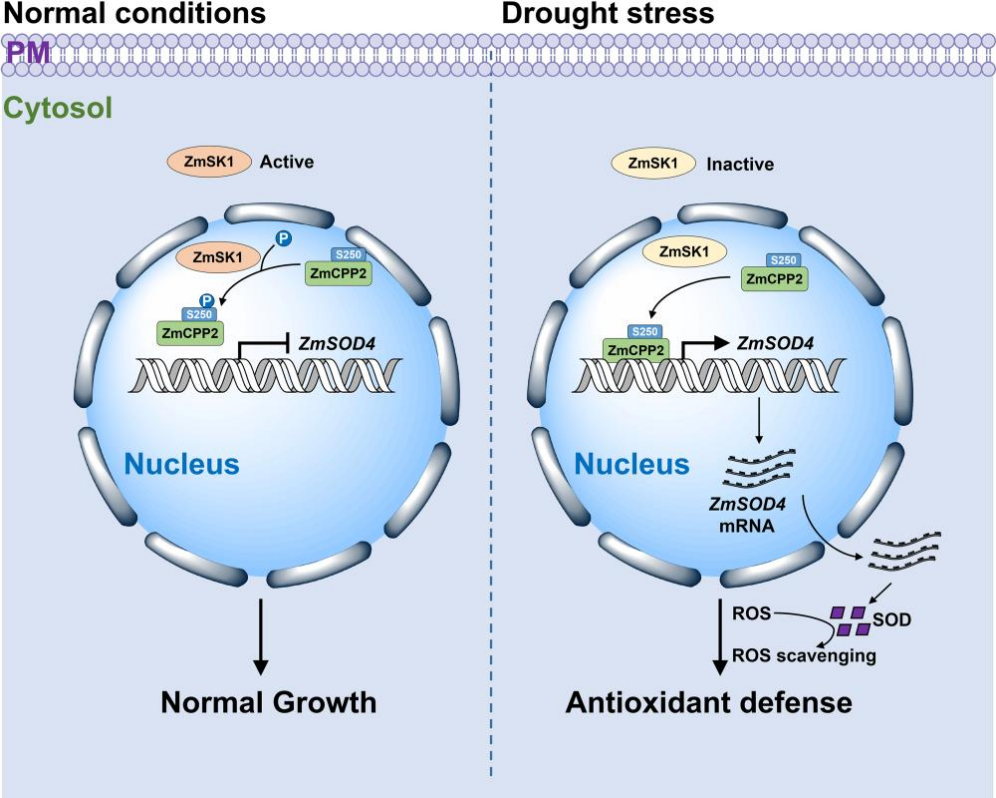
Schematic representation of the ZmSK1-ZmCPP2-ZmSOD4 module in mediating maize drought stress. Under normal conditions, ZmSK1 phosphorylates ZmCPP2, thereby inhibiting its binding to the *ZmSOD4* promoter and repressing *ZmSOD4* transcription. Under drought stress, ZmSK1 activity is inhibited, preventing the phosphorylation of serine 250 in ZmCPP2. The nonphosphorylated ZmCPP2 binds to the *ZmSOD4* promoter, activating *ZmSOD4* expression and improving maize drought tolerance. Reprinted from Xiang et al. (2025), Figure 10.

## Dive Curated Terms

The following phenotypic, genotypic, and functional terms are of significance to the work described in this paper:

CPP Gramene: ostta09g04220
CPP Araport: ostta09g04220

glycogen CHEBI: CHEBI:28087

superoxide CHEBI: CHEBI:18421
